# Comparison of predictive models for hepatitis C co-infection among HIV patients in Cambodia

**DOI:** 10.1186/s12879-020-4909-z

**Published:** 2020-03-12

**Authors:** Jozefien Buyze, Anja De Weggheleire, Johan van Griensven, Lutgarde Lynen

**Affiliations:** Department of Clinical Sciences, Institute of Tropical Medicine, Nationalestraat 155, Antwerpen, 2000 Belgium

**Keywords:** HCV-HIV co-infection, Spiegelhalter-Knill-Jones, Logistic regression, CART

## Abstract

**Background:**

Hepatitis C virus (HCV) infection is a major global health problem. WHO guidelines recommend screening all people living with HIV for hepatitis C. Considering the limited resources for health in low and middle income countries, targeted HCV screening is potentially a more feasible screening strategy for many HIV cohorts. Hence there is an interest in developing clinician-friendly tools for selecting subgroups of HIV patients for whom HCV testing should be prioritized. Several statistical methods have been developed to predict a binary outcome. Multiple studies have compared the performance of different predictive models, but results were inconsistent.

**Methods:**

A cross-sectional HCV diagnostic study was conducted in the HIV cohort of Sihanouk Hospital Center of Hope in Phnom Penh, Cambodia. We compared the performance of logistic regression, Spiegelhalter-Knill-Jones and CART to predict Hepatitis C co-infection in this cohort. We estimated the number of HCV co-infections that would be missed. To correct for over-optimism, the leave-one-out bootstrap estimator was used for estimating this quantity.

**Results:**

Logistic regression misses the fewest HCV co-infections (8%), but would still refer 98% of HIV patients for HCV testing. Spiegelhalter-Knill-Jones (SKJ) and CART respectively miss 12% and 29% of HCV co-infections but would only refer about 30% for HCV testing.

**Conclusions:**

In our dataset, logistic regression has the highest log-likelihood and smallest proportions of HCV co-infections missed but Spiegelhalter-Knill-Jones has the highest area under the ROC curve. The likelihood ratios estimated by Spiegelhalter-Knill-Jones might be easier to interpret for clinicians than odds ratios estimated by logistic regression or the decision tree from CART. CART is the most flexible method, and no model has to be specified regarding presence of interactions and form of the relationship between outcome and predictor variables.

## Background

Hepatitis C virus (HCV) infection is a major global health problem. 71 million people are chronically infected and HCV-attributable mortality kept rising the last 20 years to 495.000 annual deaths in 2015 [1]. Until recently, treating HCV was complex, not affordable, poorly successful and not considered for programming in low and middle income countries (LMIC). Recently, with the advent of affordable generic HCV Direct Acting Antivirals this changed. The new global HCV cascade targets—90% of infected diagnosed and 80% of diagnosed treated by 2030—reflect this paradigm shift [2]. To allow timely scale up of treatment, efficient HCV testing strategies will thus be crucial. Less than 15% of those living with hepatitis C know their status, with even lower proportions in LMIC [3]. WHO guidelines recommend screening all people living with HIV for hepatitis C. For the general population, the recommendation is tailored according to prevalence; universal screening if prevalence above 2 or 5%, and targeted screening if lower [4]. Considering the limited resources for health in LMIC, and recent data indicative of low-to-intermediate HCV/HIV co-infection rates among HIV populations without specific risk profile [5, 6], targeted HCV screening is potentially a more feasible and cost-effective screening strategy for many HIV cohorts in LMIC (except for HIV populations with higher risk profile, as men having sex with men, and people who use drugs), especially in this initial phase of HCV care scale-up. Simple tools or scores to guide targeted screening, other than birth-cohort screening, do not exist. However, HCV screening based on older age as sole criterion might be too restrictive for LMIC where drivers of generalized HCV exposure were often removed much later or only partially [7].

Hence there is an interest in developing other, more sensitive, but clinician-friendly tools for selecting subgroups of HIV patients for whom HCV testing should be prioritized, i.e. in predicting active HCV co-infection (defined as HCV-RNA detected). When developing a predictive model, multiple items might be of prognostic value. Since these items are typically correlated, the predictive model should take this dependency into account. Logistic regression [8] is widely used when the outcome is a binary variable. However, several other approaches have been developed, e.g. classification and regression trees (CART) [9] and the Spiegelhalter-Knill-Jones (SKJ) approach [10]. Several studies have compared the performance of different predictive models, but results were inconsistent [11, 12]. While the SKJ method requires all predictors to be categorical, the logistic regression model and CART are able to incorporate continuous predictors too. Another advantage of CART is that it does not require a pre-defined underlying relationship between the predictors and the outcome. The goal of this paper is to compare the performance of these three methods to predict HCV co-infection in a cohort of Cambodian HIV-infected patients.

## Methods

### Data source

We compared the performance of the predictive models on a dataset of a cross-sectional HCV diagnostic study conducted in the HIV cohort of Sihanouk Hospital Center of Hope (SHCH) in Phnom Penh, Cambodia (clinicaltrials.gov NCT02361541) [5]. The information on potential predictors (by history-taking, physical examination and laboratory testing) was collected prospectively following a pre-specified study protocol, and whilst results of HCV diagnostic testing were yet unknown. In total, 3045 adult HIV patients were enrolled, of whom 106 with a current HCV co-infection (i.e. HCV-RNA detected). We built the predictive models including the following items: age (years), gender (female/male), platelet count (×10^9^ cells/L), aspartate aminotransferase (AST, IU/L), alanine aminotransferase (ALT, IU/L), AST-to-platelet ratio index (APRI), having diabetes mellitus (yes/no), any of the following symptoms: fatigue, myalgia/arthralgia, anorexia/weight loss (yes/no), presenting generalized pruritus without obvious skin lesions (yes/no), having a household member and/or partner with liver disease (yes/no), and poor CD4 recovery on ART, i.e. CD4 below 200 after 3 years or more on ART (yes/no).

### Performance of predictive model

In this setting, we wanted to select a subset of HIV patients at higher risk of HCV co-infection for whom HCV testing should be prioritized. In absence of a well-established threshold for HCV testing, we considered the harm/benefit of testing and not testing (at patient and public health level). We intended a lower threshold than the WHO recommended threshold (2-5% depending on resource availability) for HCV testing in the general population [4], because HIV populations in resource-constrained settings remain at higher risk of advanced HCV disease as they have often started antiretroviral therapy late or with less optimal regimens. A 1% probability threshold for the decision rule (i.e. giving false negatives much more weight than false positives) seems low enough as the risk score, if easily applicable, can be repeated yearly. Hepatitis C treatment is in most cases not urgent. Hence our aim was to build a prediction model where the probability of HCV co-infection in the group who is classified as negative is smaller than 1%. To compare performance of the prediction models obtained with the different methods, we estimated the log-likelihood, the area under the ROC curve, the number of HCV co-infections that would be missed, the sensitivity, specificity, positive and negative predictive value. To correct for over-optimism, the leave-one-out bootstrap estimator [13] was used. Furthermore, we compared the proportion of participants who would be referred to HCV testing.

### Logistic regression

The logistic regression model is

$$\log\left(\text{odds}\right)=\alpha+\beta_{1}x_{1}+\cdots+\beta_{p}x_{p}$$

where *x*_1_,⋯,*x*_*p*_ are the different predictors. The coefficients *β*_*i*_ represent the adjusted log odds ratio (OR) for each difference of one unit in *x*_*i*_. The intercept, *α*, is the log odds when all predictors are equal to zero. The logistic regression model can include continuous, binary and categorical predictors. Missingness was added as a factor level to variables for which there are missing values.

A logistic regression model was fitted with all candidate predictors as independent variables. Because of sparse data, Firth correction was applied. The predictor score was calculated by rounding $\hat {\beta }_{1}x_{1}+\cdots +\hat {\beta }_{p}x_{p}$. A cutoff was chosen as the minimal value such that in the group of subjects with this score, the proportion of subjects with HCV co-infection was larger than 1%. All subjects with a score of at least this cutoff were classified as needing HCV testing.

### Classification and regression trees

Classification and regression trees (CART) use recursive binary partitions to divide the predictor space into a set of subregions [9]. More specifically, the covariate space of the root node is split into two child nodes, based on the predictor and cutoff that yields the largest decrease in impurity (i.e. less heterogeneity in outcome within each node). Next, one of these child nodes is split into two more nodes. This procedure is repeated under the following conditions: a node has to contain at least 20 observations to be considered for splitting and a terminal leaf has to contain at least 7 observations. Since this process likely over-fitted the data, the tree was pruned to a smaller sub-tree. A penalty is added to the error of the tree, relative to the size of the tree. A sequence of trees was fitted with each time a different cost-complexity parameter (i.e. penalty for the size of the tree). The smallest tree whose error lies within one standard error of the minimal error over the sequence of trees was selected. The weight for false negatives was chosen so that the proportion of true HCV co-infections in the group who are classified as negative is smaller than 1%. For each split a surrogate variable is identified which approximates the split using another predictor variable. Any observation which is missing the split variable is then classified using the surrogate variable [14].

### Spiegelhalter-Knill-Jones

The Spiegelhalter-Knill-Jones (SKJ) approach adapted by Berkley et al. [10, 15] estimates likelihood ratios. Because the SKJ approach requires binary predictor variables, the continuous candidate predictors were dichotomized using the cutoff which maximizes the Youden index. In a first step, unadjusted likelihood ratios (LR) for all candidate predictors are estimated, and the predictors with an unadjusted LR ≥2 or ≤0.5 are included in a next step, in the multivariable logistic regression model:

$$\log\left(\text{odds}\right)=\alpha+\beta_{1}w_{1}+\cdots+\beta_{p}w_{p}$$

where *w*_*i*_ is the crude log positive/negative LR for positive/negative test results respectively. The adjusted likelihood ratios (aLR) are then given by

$$\begin{array}{*{20}l} {\mathrm{aLR+}}_{\mathrm{i}} &= \exp{\left({\beta }_{i}\times \log {\left({\mathrm{LR+}}_{\mathrm{i}}\right)}\right)}\\ {\mathrm{aLR-}}_{\mathrm{i}} &= \exp{\left(\beta_{i}\times \log {\left({\mathrm{LR-}}_{\mathrm{i}}\right)}\right)} \end{array} $$

where *β*_*i*_ is the shrinkage factor from crude LR to adjusted LR. The predictors with an aLR ≥1.5 or ≤0.67 were selected for the final predictive model. The aLRs were transformed to their natural logarithm, and rounded to the nearest integer to calculate the score (relative weight) of each predictor. By summing the scores of all predictors presented by a patient the total predictor score for each patient was obtained. A value of 0 was assigned to missing data, assuming that a missing value is not predictive. A cutoff was chosen as the minimal value such that in the group of subjects with this score, the proportion of subjects with HCV co-infection was larger than 1%. All subjects with a score of at least this cutoff were classified as needing HCV testing.

Statistical analysis was performed in Stata 15.1 [16] and R 3.5.0 [17].

## Results

A total of 3045 ambulatory HIV patients of Sihanouk Hospital Center of Hope were included. Their median age was 43 years (interquartile range (IQR): 36–48), 43% were male patients, 98% were on antiretroviral therapy (ART), and 1% (N=31) reported past or current sex work, being homosexual, or a history of injecting drug use. In this cohort, 106 patients had a detectable HCV-RNA (our outcome of interest), but none among the above-mentioned 31 HIV patients with higher risk profile. Distribution of the candidate predictors in the cohort and the missing values are further specified in Table 1.

**Table 1:** Patient characteristics

Characteristics	Missing values	*n*=3045
Male, *n* (%)	0	1307 (42.9)
Age, years, median (IQR)	0	42.5 (36.3–48.1)
Poor CD4 recovery on ART, *n* (%)	13	117 (4.0)
ALT, IU/L, median (IQR)	0	28 (20–43)
AST, IU/L, median (IQR)	0	26 (21–36)
Platelets, ×10^9^ cells/L, median (IQR)	0	266 (221–312)
APRI, median (IQR)	0	0.29 (0.21–0.41)
Fatigue, myalgia/arthralgia, or anorexia/weight loss, *n* (%)	0	301 (9.9)
Generalized pruritus, *n* (%)	0	120 (3.9)
Diabetes mellitus, *n* (%)	6	113 (3.7)
Partner or household member with liver disease, *n* (%)	10	185 (6.1)

### Predictive models: logistic regression, cART, spiegelhalter-Knill-Jones

The adjusted odds ratios from the logistic regression model are shown in Table 2. A higher age, ALT, APRI and having a partner or household member with liver disease increase the probability of HCV co-infection, while higher platelet levels and being a male decrease the probability of HCV co-infection. The number of observed HCV co-infections for each score are shown in Table 3. A score of −2 is the lowest score for which the proportion of subjects who are HCV co-infected is larger than 1%. Thus all subjects with a prediction score of −2 or higher would be referred to HCV screening.

**Table 2:** Logistic regression

Predictor	Adjusted OR	p-value
Age (per 10 years)	1.66	<0.001
Male gender	0.55	0.008
Platelets (per 10×10^9^ cells/L)	0.91	<0.001
AST (per 10 IU/L)	0.93	0.078
ALT (per 10 IU/L)	1.09	0.037
APRI	1.30	0.029
Diabetes mellitus		
Yes	2.15	0.052
Missing	22.89	0.002
Fatigue or myalgia/arthralgia	1.35	0.33
or anorexia/weight loss		
Generalized pruritus	1.83	0.16
Household member and/or		
partner with liver disease		
Yes	3.68	<0.001
Missing	1.08	0.96
Poor CD4 recovery on ART		
Yes	0.99	0.99
Missing	1.90	0.69

**Table 3:** Prediction score

score	no HCV co-infection	HCV co-infection	Total
logistic regression			
-6	1	0	1
-5	3	0	3
-4	6	0	6
-3	44	0	44
-2	302	4	306
-1	1116	8	1124
0	1022	33	1055
1	363	30	393
2	63	14	77
3	13	5	18
4	6	9	15
5	0	2	2
8	0	1	1
Spiegelhalter-Knill-Jones			
-2	1167	7	1174
-1	936	9	945
0	245	11	256
1	329	16	345
2	194	28	222
3	61	24	85
4	6	8	14
5	1	3	4

**Fig. 1:**
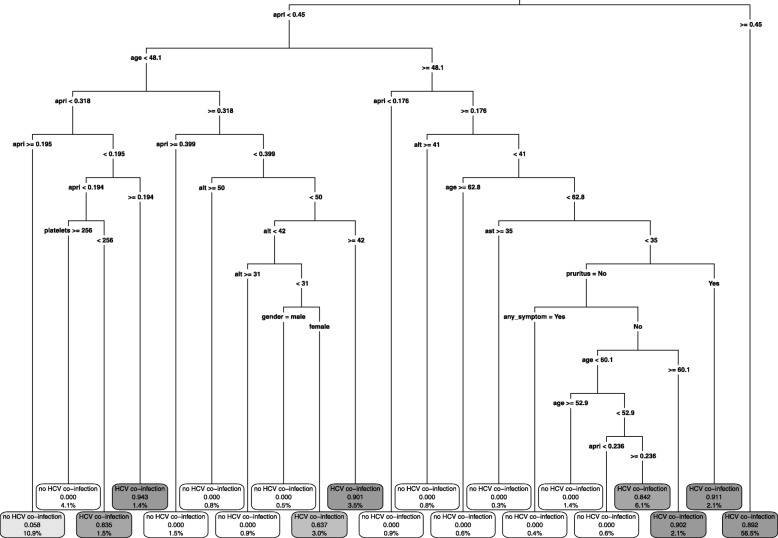
CART. The leaves show the predicted HCV co-infection status, the probability of HCV co-infection and the percentage of observations in the leaf

In CART, to ensure that the proportion of true HCV co-infections in the group who are classified as negative is smaller than 1%, the selected weight for false negatives was 58. The predictors used in the tree (Fig. 1) are: age, gender, platelets, AST, ALT, APRI, any of fatigue, myalgia/arthralgia, anorexia/weight loss and generalized pruritus. Of the 106 subjects with HCV co-infections, 105 would be referred for HCV screening, compared to 839 of the 2939 subjects without HCV co-infection.

The unadjusted and adjusted likelihood ratios of the candidate predictors resulting from the Spiegelhalter Knill-Jones method are reported in Table 4. The predictors retained for the score were: age ≥50 years, platelets <200×10^9^ cells/L, AST ≥30 IU/L, APRI ≥0.45, diabetes mellitus, generalized pruritus and household member and/or partner with liver disease (Table 4). The number of observed HCV co-infections for each score are shown in Table 3. A score of 0 is the lowest score for which the proportion of subjects who are HCV co-infected is larger than 1%. Thus all subjects with a prediction score of 0 or higher would be referred to HCV screening.

**Table 4:** Spiegelhalter-Knill-Jones

Predictor	Unadjusted LR		Adjusted LR		score
	LR+	LR-	aLR+	aLR-	
Age ≥50 years	2.25	0.71	2.18	0.72	+1
Male gender	0.99	1.01	-	-	
Platelets <200×10^9^ cells/L	3.46	0.62	1.69	0.82	+1
AST ≥30 IU/L	2.21	0.28	1.48	0.53	−1
ALT ≥40 IU/L	2.33	0.49	-	-	
APRI ≥0.45	3.88	0.33	2.42	0.48	+1/−1
Diabetes mellitus	3.76	0.90	2.14	0.94	+1
Fatigue or myalgia/arthralgia	2.11	0.88	-	-	
or anorexia/weight loss					
Generalized pruritus	2.61	0.94	2.04	0.95	+1
Household member and/or	3.21	0.87	3.62	0.85	+1
partner with liver disease					
Poor CD4 recovery on ART	1.34	0.99	-	-	

### Predictive performance of the different models

The predictive performance of the different models is shown in Table 5. Logistic regression obtains the highest log-likelihood and misses the fewest HCV co-infections, but would still refer 98% of HIV patients for HCV testing. Spiegelhalter-Knill-Jones has a higher area under the ROC curve and misses fewer HCV co-infections than CART but has a lower specificity and positive predictive value. Both methods would refer about 30% for HCV testing. This would yield a high cost reduction compared to testing all HIV patients for HCV.

**Table 5:** Comparison of predictive performance

	Logistic regression	CART	Spiegelhalter-Knill-Jones
log-likelihood	-201.3	-267.8	-209.6
Area under ROC curve	74.6%	73.4%	81.9%
Porportion of HCV	7.8%	28.8%	12.1%
co-infections missed			
Sensitivity	92.2%	71.2%	87.9%
Specificity	22.1%	73.3%	50.5%
Positive predictive value	4.1%	8.7%	5.9%
Negative predictive value	98.7%	98.6%	99.2%
Proportion for whom	98.2%	31.0%	30.4%
HCV testing is needed			

## Discussion

In our dataset, logistic regression has the highest log-likelihood and smallest proportions of HCV co-infections missed but refers more subjects for HCV screening. Depending on the specific setting, a balance needs to be made between the number of HCV co-infections missed and the number of HCV tests to perform. In general for a triage test (like a clinical scoring system), a higher sensitivity is preferred, and the specificity is determined by the resources available. A limitation of our study is that our goal was not to compare the predictive performance of logistic regression, CART and SKJ in general, but only in this specific case of predicting HCV co-infection in the study population of Cambodian HIV-infected patients. Our findings may not be generalizable to other outcomes. Also generalizability of the different derived models for our outcome (HCV co-infection) could not be ascertained, this would require further external validation.

When the aim is to predict a binary outcome, logistic regression is widely used. The association of each predictor with the outcome is expressed as an adjusted odds ratio, which might be difficult for clinicians to interpret. However if the goal is to build a prediction model, the interpretation of the relationship between predictor and response is probably not of interest. Furthermore, for classification, the score needs to be calculated, which is not very user-friendly. Although an app could be developed that calculates this score based on values of the predictor variables. The usefulness of a clinical prediction rule is also determined by its ease of use. The SKJ method estimates adjusted likelihood ratios, positive or negative if key predictors are present or absent, and this more nuanced information is preferred above odds ratios by clinicians. Moreover the score can be easily calculated, as a sum of integers. Also CART results in a decision tool that can be easily applied in clinical practice. However the relationship between predictor and reponse is harder to interpret than with logistic regression or SKJ.

In logistic regression, missing values were considered as an extra level of the covariate factor. However this approach is known to be biased, even when missingness is completely at random. Other methods to handle missing data are available, like multiple imputation, but all of them depend on untestable assumptions. They are also more complex and would yield a score not feasible to apply in clinical practice. On the other hand, missing values are naturally handled by SKJ making the assumption that a missing value is not predictive of the outcome (the score corresponds to 0 and does not affect the prediction in confirmation or exclusion). Using CART, for subjects with a missing value for a splitting variable a surrogate split is used.

The SKJ corrects for confounding, but does not allow interactions between predictors, and the shrinkage used is similar for a negative or a positive test result, i.e. LR+ and LR-. Interactions can be included in the logistic regression model, but they have to be specified. In practice often only two-way interactions are included, if any. Because of the way they are built, CART naturally includes higher-order interactions, derived from the data. In that sense CART is the most flexible method, and no model has to be specified.

The performance of CART can be improved by using random forests or boosted trees [13]. Both methods aggregate information from multiple decision trees, developed on different bootstrap samples. Although their predictive performance surpasses that of a single tree, both random forests and boosted trees do not yield a simple decision rule. Hence we did not consider them in this paper since our aim was to develop a prediction rule that can be easily applied in clinical practice.

## Conclusions

When the goal is to predict a binary outcome, often logistic regression is chosen as method to build a prediction score. However other methods like SKJ and CART may perform better and should be considered. More research is needed on how to select the best prediction method in a certain setting.

## Data Availability

The data supporting the findings of this study are retained at the Institute of Tropical Medicine, Antwerp and will not be made openly accessible due to ethical and privacy concerns. Data can however be made available after approval of a motivated and written request to the Institute of Tropical Medicine at ITMresearchdataaccess@itg.be.

## Abbreviations

aLR: adjusted likelihood ratio
ALT: alanine aminotransferase
APRI: AST-to-platelet ratio index
ART: antiretroviral therapy
AST: aspartate aminotransferase
CART: classification and regression trees
HCV: Hepatitis C virus
IQR: interquartile range
LMIC: low and middle income countries
LR: likelihood ratio
OR: odds ratio
SHCH: Sihanouk Hospital Center of Hope
SKJ: Spiegelhalter-Knill-Jones
